# Memristance and transmemristance in multiterminal memristive systems

**DOI:** 10.1038/s41598-026-35671-7

**Published:** 2026-01-14

**Authors:** Gianluca Milano, Davide Pilati, Fabio Michieletti, Alessandro Cultrera, Carlo Ricciardi, Enrique Miranda

**Affiliations:** 1 Advanced Materials Metrology and Life Sciences Division, INRiM (Istituto Nazionale Di Ricerca Metrologica), Strada Delle Cacce 91, 10135 Torino, Italy; 2 Department of Applied Science and Technology, Politecnico Di Torino, C.So Duca Degli Abruzzi 24, 10129 Torino, Italy; 3 Quantum Metrology and Nanotechnologies Division, INRiM (Istituto Nazionale Di Ricerca Metrologica), Strada Delle Cacce 91, 10135 Torino, Italy; 4 Departament d’Enginyeria Electrònica, Universitat Autònoma de Barcelona (UAB), 08193 Cerdanyola del Vallès, Spain

**Keywords:** Engineering, Materials science, Mathematics and computing, Nanoscience and technology, Physics

## Abstract

Memristive devices represent promising building blocks for the development of next-generation memory technologies, computing architectures, and neuromorphic systems. In addition to conventional two-terminal memristive circuits and crossbar array structures, multiterminal memristive systems, where emergent behaviours arise from the mutual interaction of numerous memristive elements, have been explored for neuromorphic data processing and computing applications. In this work, we extend the concept of two-terminal memristive devices to generic multiterminal memristive systems. Beyond its ability to describe the specific case of crossbar arrays, the proposed theoretical framework is also applicable to more complex systems such as self-organizing memristive networks, whose internal state dynamics depend not only on time-varying input signals but also on the spatial distribution of the stimulated terminals. After discussing the notion of *memristance* in multiterminal devices as the evolution of the system “seen” from the stimulating terminals, we demonstrate that the two-terminal memristive framework can be generalized to the concept of *transmemristance* when additional, non-stimulating electrodes are used to monitor the system’s evolution. Providing a connection between circuit theory and network science, these concepts are investigated both analytically and experimentally using a theoretical memristive graph model and an experimental memristive system based on self-organizing nanowire networks.

**Supplementary Information:**

The online version contains supplementary material available at 10.1038/s41598-026-35671-7.

## Introduction

Since the experimental demonstration of the two-terminal memristive device^1^, where the theoretical concept of memristor^2,3^ was associated with resistive switching phenomena in nanoionic structures^4^, these devices has been considered a potential breakthrough in electronics. The key element behind the memristive behaviour is that its resistance state depends on the history of electrical stimulation. For this reason, these devices have been widely considered building blocks for the realization of next-generation memories as well as for the development of novel computing architectures, hardware accelerators for artificial intelligence, hardware implementations of artificial neural networks, and neuromorphic systems^5–11^. To accomplish these tasks, two-terminal devices are often organized in grid-like arrays to form the so-called crossbar architecture^12^. However, in parallel with advancements in memristive systems based on two-terminal memristive cells, unconventional memristive architectures consisting of a large number of interacting memristive elements forming complex systems have been explored.^13^ In these memristive systems, the interest lies in the collective response of the electrical network rather than in the isolated conduction characteristic of each memristive element. It is in this context that their resultant behaviour has been investigated through experiments and simulations demonstrating the implementation of a wide range of computing tasks, including pattern recognition, speech recognition, nonlinear transformation, and time-series prediction^14–26^. Furthermore, it has been demonstrated that networks of memristive cells can be exploited for maze solving^27^ and for solving shortest-path optimization problems^28^. Similarly to what occurs in two-terminal memristive cells, the resistance state of these complex memristive systems depends on the history of electrical stimulation. In these systems, which differ from memristive devices where a modulatory signal or additional electrodes are used to modify or influence the memristive transport properties of an otherwise conventional two-terminal device,^29,30^ the ability to stimulate the network at different locations through multiple electrical terminals enables the realization of multiterminal memristive systems whose internal state evolution also depends on the spatial position of the stimulation.^31^. Also, the multiterminal configuration enables observation of the evolution of the internal state of resistance through multiple terminals, i.e. according to different electrical “points of view”^32^ Importantly, approaches developed for analyzing crossbar arrays, where the ordered arrangement of devices simplifies the analysis, cannot be directly generalized to unconventional multiterminal architectures. In this context, a comprehensive generalization of the two-terminal memristive concept to generic multiterminal systems, encompassing both the specific case of crossbar arrays and more complex configurations such as self-organizing memristive networks, is still lacking.

In this work, we extend the concept of a two-terminal memristive device to a generic multiterminal memristive system where the internal state relies on the history of applied spatiotemporal stimulation and memristive behaviour can be observed across multiple terminal pairs. In these systems, the evolution of the electrical distance between two terminals can be interpreted as a *memristive distance*. In addition, we show that these systems allow the extension of the two-terminal memristive concept, where the same terminals are exploited both to stimulate and read the internal state, to the multiterminal transmemristance concept, where the evolution of the internal state can be tracked also through terminals that are not being directly stimulated. These concepts are discussed by exploiting a theoretical memristive graph and an experimental multiterminal memristive system based on self-organizing nanowire networks as examples.

###  Two-terminal memristive systems

The definition of a memristive system adopted here follows Chua’s generalization of the memristor to nonlinear dynamical systems, highlighting its behavioural nature arising from the evolution of an internal state variable under external excitation^33^. According to Chua’s theory, a memristive system is described by one equation for the memory state and one equation for the transport characteristic, both expressed respectively as:

1 $$\frac{dx}{dt}=F\left(x,u, t\right)$$

2 $$M\left(x,u, t\right)u=y$$

where $x$ represents the memory state of the device, $u$ is the voltage (or current), $t$ is the time, $y$ the output current (or voltage) of the two-terminal structure, and $M$ its memductance (or memristance). Physically, in redox-based memristive devices, Eq. (1) is associated with the electric field-driven displacement of ions or vacancies, depending on the type of switching material, while Eq. (2) represents the electron flow. In this context, it is worth remarking that Eq. (2) implies a linear relationship between $u$ and $y$ that can represent only a first-order approximation in some physical devices where this relationship can be regulated by more complex dependencies. For example, a memdiode model considering a hyperbolic sine dependence for the current–voltage characteristic which becomes linear in the low voltage region have been reported^34^. Note that the function $F$ defines the system dynamics and can depend on external stimulation, memory state of the device, and time. Different formulations of $F$ have been reported to describe the dynamics of both non-volatile and volatile memristive systems. This includes flux-charge models^35^, formation and dissolution of atomic-size gaps^36^, behavioural trajectories^37^, behavioural models based on a hysteresis operator (with explicit dependence on $t$ to model fatigue profiles)^38^, stochastic models including quantum conductance effects^39^, potentiation-depression rate-balance Equation^40^, etc.

### Multiterminal memristive systems

The concept of two-terminal memristor and, more generically, the concept of two-terminal memristive system^33^, can be extended to the concept of *multiterminal memristive system*. In the context of this work, we refer to the concept of multiterminal memristive system as a generalized form of dynamical system with multiple electrical inputs/outputs whose internal state depends on internal state variables evolving according to the history of input (multiple) signals. According to this definition, memristive crossbar arrays^12^ as well as multiterminal devices based on self-organizing memristive networks^13^ can be considered as multiterminal memristive systems.

Let us consider a multiterminal memristive system composed of $N$ terminals. In general, not all terminals of the system can be contacted (because of limitations in the experimental setup or due to the inability to correctly identify a particular terminal); therefore, we define a subset of ${N}_{A}$ terminals that are accessible from the outside. Among these ${N}_{A}$ accessible terminals, we further define a subset of $n$ terminals that are held at well-defined potentials, *i.e.* the terminals that are not floating ($n$ $\le$ ${N}_{A}$ $\le$ *N)*. By considering these *n* terminals with known potentials, the system can be generically described by two coupled equations: one governing the evolution of its internal memory state and another (matrix) equation describing the electronic transport behaviour:

3 $$\frac{d\mathcal{x}}{dt}=\mathcal{F}(\mathcal{x},\mathcal{u},t)$$

4 $$\left[\begin{array}{cccc}{M}_{11}& {M}_{12}& \cdots & {M}_{1n}\\ {M}_{21}& {M}_{22}& & \\ \vdots & & \ddots & \\ {M}_{N1}& & & {M}_{nn}\end{array}\right]\left[\begin{array}{c}{u}_{1}\\ {u}_{2}\\ \vdots \\ {u}_{n}\end{array}\right]=\left[\begin{array}{c}{y}_{1}\\ {y}_{2}\\ \vdots \\ {y}_{n}\end{array}\right]$$

where Eq. (4) can be rewritten in the matrix form as:

5 $$\mathcal{M}\left(\mathcal{x},\mathcal{u}, t\right)\mathcal{u}= \mathcal{y}$$

In Eq. (5), ${\boldsymbol{x}}$ is the memory state of the multiterminal system representing in some contexts the collective state of the system and in others the collection of individual states, $\mathcal{u}$ are voltages (or currents), $\mathcal{y}$ are currents (or voltages) at terminals of the multiterminal system, and $\mathcal{M}$ is defined as the memductance (or memristance) matrix of the system. As discussed before in case of a two-terminal memristive device, also in this case the linear relationship between $\mathcal{u}$ and $\mathcal{y}$ provided by Eq. (5) can represent only a first-order approximation of more complex dependencies between these quantities. The function $\mathcal{F}$, whether or not its analytical expression is known, defines the specific dynamics of the multiterminal system, which are determined by the underlying physicochemical mechanisms responsible for the memristive behaviour and depend on external spatiotemporal (multiterminal) stimulation, the internal state of the system, and possible other factors. Importantly, it should also be noted that, in general, the function $\mathcal{F}$ may depend on the specific subset of terminals being stimulated; that is, $\mathcal{F}$ is not a unique function of the system but may vary with the specific stimulation configuration.

According to the previous discussion, the matrix $\mathcal{M}$, which electrically describes the system, is constructed by considering the subset of $n$ nodes held at well-defined potentials. Consequently, $\mathcal{M}$ is an $n \times n$ matrix. Each element of $\mathcal{M}$ may depend on the system’s memory state ${\boldsymbol{x}}$, representing its collective internal configuration or a given individual state, on the set of voltages or currents applied to specific terminals of the multiterminal structure, and on time. Notice also that the signs of the elements of $\mathcal{M}$ depend on the chosen reference directions for the terminal currents. The diagonal entries of the memductance matrix $\mathcal{M}$ correspond to the self-memductances, while the off-diagonal entries represent the mutual memductances between terminals, where ${\mathcal{M}}_{ij}= {\mathcal{M}}_{ji}$ under the assumption of a reciprocal network. The self-memductance coincides with the inverse self-memristance between two selected terminals if and only if all other terminals are left floating. Note that Eq. (4) represents the extension of Eq. (2) to a multiterminal system.

**Fig. 1:**
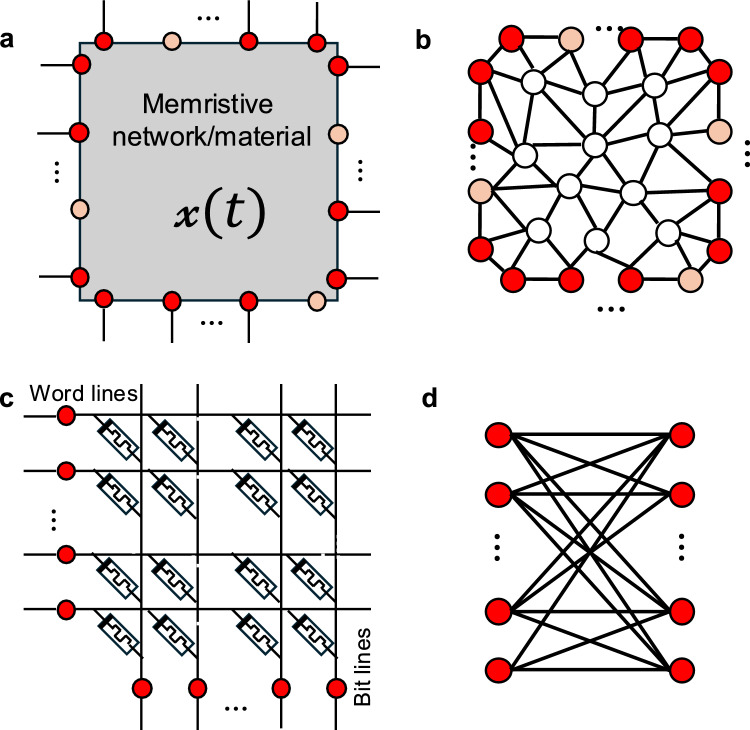
Multiterminal memristive systems. (**a**). Conceptual schematization of a generic multiterminal memristive system where the internal state of the system $x(t)$ rely on the interaction of the system with the environment through accessible terminals. Among represented accessible terminals ${N}_{A}$, red terminals are the subset of $n$ terminals held at well-defined potentials (not floating), while orange terminals are the subset of ${N}_{A}-n$ terminals that are left floating. (**b**). Representation of a generic multiterminal memristive system through graph theory that, besides being characterized by terminals ${N}_{A}$ accessible nodes that are held at well-defined potentials (red nodes) or are left floating (orange nodes). (**c**). Conceptual schematization of a crossbar architecture as a multiterminal memristive system with word lines and bit lines and d. corresponding representation of the multiterminal crossbar architecture as a bipartite graph. In general, in this architecture, voltages are applied to word lines, while bit lines are connected to ground. This means that in this case all accessible nodes are (at least in the conventional configuration) at a well-defined potentials (${N}_{A}$= $n$).

Figure 1a presents a conceptual schematic of a multiterminal memristive system with ${N}_{A}$ accessible terminals (or nodes) that allow the system to interact with its environment. These accessible terminals can, in principle, represent a subset of the total *N* terminals, since additional internal terminals may exist within the memristive network or material, which is here represented as a black box. Depending on the specific system and the evolution of its interaction with the environment, the accessible terminals may dynamically change their function over time. For example, an input/output terminal can become a read terminal (and vice versa, as in Ref.^32^), or a terminal may simultaneously perform multiple functions (e.g., acting as both input and output, as in Ref.^15^). In Fig. 1a, red nodes represent the subset of $n$ nodes held at well-defined potentials, while orange nodes correspond to accessible terminals that are left floating (and thus do not contribute to the definition of $\mathcal{M}$). Voltages at the $n$ terminals are defined with respect to an external reference node (ground), which is not shown in the figure. The multiterminal memristive system depicted as a black box in Fig. 1a can be based either on a complex network of discrete memristive elements or on a memristive material exhibiting spatially continuous memristive properties. It is worth noting that a sufficiently large network of interacting discrete memristive elements can be approximated as a spatially continuous memristive medium^41^. Within this framework, the multiterminal memristive system can be mathematically represented as a graph in which edges correspond to individual memristive elements (memristive graph, Fig. 1b). In addition to the accessible nodes that interact with the environment, either held at well-defined potentials (red nodes in Fig. 1b) or left floating (orange nodes), the graph may also include internal nodes (white nodes), which represent internal connections between memristive elements that are not directly accessible from outside the system. According to Kirchhoff’s laws, both internal nodes and accessible nodes that are left floating are characterized by zero net current; that is, for a generic internal or floating node $n$, $\sum {I}_{n }=0$. The memristive graph representation can be employed either to directly map the complex network structure or to approximate the system through a parcellation approach, in which nodes represent network regions and edges describe the memristive interactions between them. This is the case, for instance, in sufficiently dense and disordered memristive nanowire networks, which can be approximated as continuous media and, through parcellation, modeled as regular grid graphs^41^. More generally, the internal structure of the multiterminal network or material can be treated as a black box characterized by effective memristive couplings between terminals, as illustrated in Fig. 1a. In this context, it is important to note that, although the dynamical evolution of the physical observables measured at the accessible terminals reflects the evolution of the internal memory state, it does not provide a complete description of it. Depending on the specific multiterminal system, the internal state may not be directly accessible or measurable.

Conventional memristive architectures based on ideal crossbar arrays with negligible wire resistances (Fig. 1c) represent a particular case of a multiterminal memristive system that can be modeled as a bipartite graph (Fig. 1d). In this configuration, the multiterminal system includes only input/output accessible terminals, with no internal nodes present ($n={N}_{A}=N$). Under conventional operating conditions, where *i)* voltages are applied to all input terminals (word lines, left column of terminals), while output terminals (bit lines, bottom row) are connected to ground, so that no accessible terminals are left floating, and *ii)* only output currents are of interest (input currents are disregarded), each element of the $\mathcal{M}$ matrix corresponds to the memductance of an individual memristive device in the array. The internal state of the entire system, $\mathcal{x}$, is thus determined by the internal states of the individual memristive elements, each evolving independently according to its own memory evolution function. Notably, in this specific case, the collective state of the system is simply the aggregation of individual device states. This condition forms the basis of the matrix–vector multiplication (MVM) operation.^42^.

### The concept of transmemristance in multiterminal memristive systems

In multiterminal systems, the evolution of the internal state can be monitored through accessible terminals (floating or not) that are not directly stimulated, providing an alternative perspective on the dynamics of the system’s internal state $\mathcal{x}(t)$. By applying an input voltage (or current) between accessible terminals $i$ and $j$, and measuring the corresponding output current (or voltage) between accessible terminals $k$ and $l$, one can define the *transmemductance* (or *transmemristance*) of a linear system as:

6 $${T}_{ij,kl}\left(\mathcal{x},\mathcal{u}\right)\Delta {u}_{ij}= {\Delta y}_{kl}$$

**Fig. 2:**
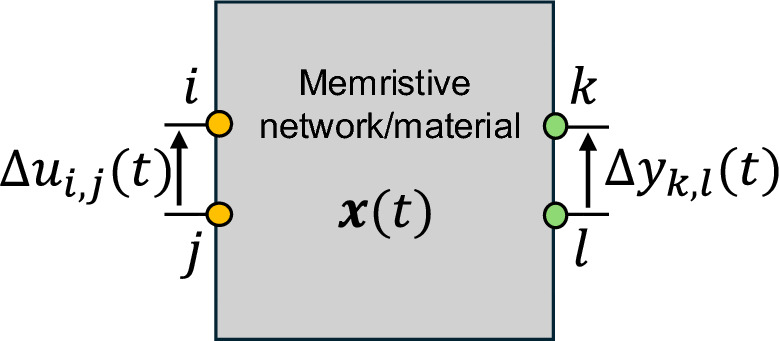
Transmemristance in a multiterminal system. a. Conceptual schematization of a multiterminal memristive system where the evolution of the internal state $x(t)$ can be observed by considering the memristive behaviour, i.e. by observing the evolution of the system in two-terminal configuration through the same terminals exploited for stimulation (while keeping all other terminals floating), or by considering the transmemristance in multiterminal configuration.

A schematic representation of a multiterminal memristive system based on a memristive network or material capable of exhibiting memristive and transmemristive responses is shown in Fig. 2.

While the transmemristance is defined as the proportionality constant that link input current with output voltage, the transmemductance is defined as the proportionality constant that link input voltage with output current. In case of transmemductance, the output current through terminals $k$ and $l$ can be evaluated by considering the internal current or the external current, giving rise to the following definitions:

*i) Internal transmemductance*: can be defined by considering as output current the current that flows inside the device produced by the transmemductance action (here we deal with internal current). In this case, it is evaluated without considering any load attached to the system. If we are considering adjacent output $k$ and $l$ node terminals of a memristive network, the internal output current is the current flowing in the edge connecting these two adjacent nodes. Note that, in general, the internal transmemductance is not a well-defined parameter if the current path between output nodes is unknown.

*ii) External transmemductance:* can be defined by considering as output current the current that flows out of the device and through a load element (or through an ammeter) that is connected between $k$ and $l$ output terminals.

In this scenario, the external transconductance serves as the practical means by which this concept can be exploited in an actual circuit implementation. It is also worth mentioning that, as the transconductance is not the reciprocal value of the transresistance^43^, the transmemductance is not the reciprocal of the transmemristance. In this scenario, the transmemristance concept provides direct insight into the dynamic coupling between terminals of the multiterminal system.

### Memristive graphs as multiterminal memristive systems

**Fig. 3:**
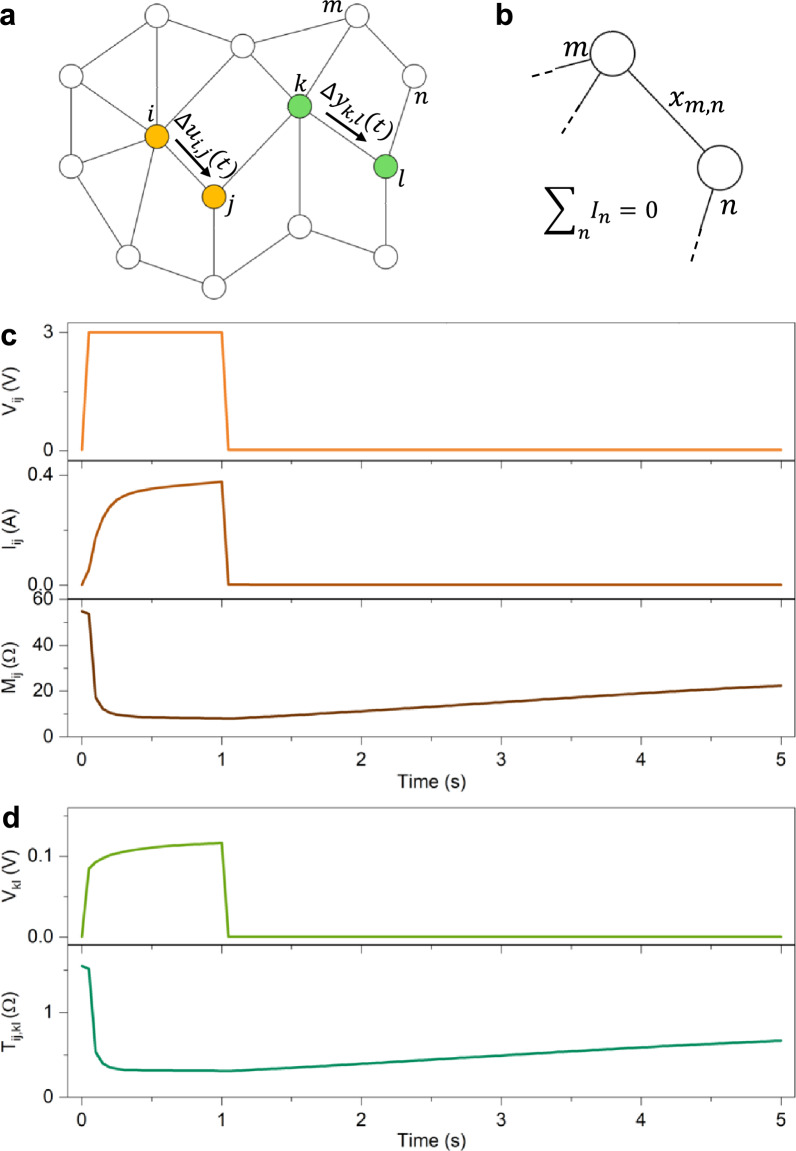
Memristance and transmemristance in a multiterminal memristive graph. (**a**). Example of a multiterminal memristive graph with accessible nodes (orange and green nodes) and internal nodes (white nodes). (**b**). Detail of the memristive interaction between $m,n$ nodes, where the internal state of the memristive edge ${x}_{m,n}$ depends on the history of electrical stimulation, and the current flowing in the $m,n$ edge is regulated by the Kirchhoff’s law. (**c**). Input pulse voltage applied in between $i$ and $j$ nodes (top panel), corresponding evolution over time of the current flowing in $i,j$ edge (middle panel), and evolution over time of the internal state of resistance (memristance) of the $i,j$ edge. d. Evolution of the voltage difference in between $k$ and $l$ nodes during stimulation of $i$ and $j$ nodes (top panel), and corresponding evolution of the transresistance over time (transmemristance) (bottom panel).

A *memristive graph* is a class of graphs characterized by memristive interactions between nodes, where the weights of the edges connecting adjacent nodes evolve over time as memristive elements according to Eqs. (1) and (2). These networks, which represent electrical circuits composed of memristive components, are complex dynamical systems whose functionalities are inherently linked to their structural topology. In a generic memristive graph, the ${N}_{A}$ accessible nodes are those that can be directly contacted for stimulating or reading the system. In this sense, accessible nodes in the graph correspond to the accessible terminals of a multiterminal memristive system. An example of a memristive graph is shown in Fig. 3a, where orange and green nodes represent accessible input and output nodes, respectively, while white nodes denote internal nodes. In the example shown here, input and output nodes are adjacent, although in the general case they may also be non-adjacent. The internal state (or weight) of a generic edge ($m,n$), denoted as ${x}_{m,n}$, depends on the history of voltage or current experienced (or externally imposed) across the nodes $m$ and *n*, where node voltages and edge currents are constrained by Kirchhoff’s laws (Fig. 3b).

Importantly, the internal state $\mathcal{x}$ of the graph also determines the Laplacian matrix $\mathcal{L}=\mathcal{D}-\mathcal{A}$, where $\mathcal{D}$ is the degree matrix, *i.e.* the matrix containing information about the (weighted) degree of each node, and $\mathcal{A}$ is the adjacency matrix, which establishes the weighted connections among all $N$ nodes of the system (both floating and non-floating). Note that the Laplacian matrix depends on the network topology and on the internal state of each edge. In electrical engineering applications, the Laplacian matrix is usually referred to as the nodal admittance matrix. The time evolution of the memristive system over time can thus be represented by the time evolution of the Laplacian matrix $\mathcal{L}(t)$. At a given time ${t}^{*}$, the node voltages and edge currents can be computed from the matrix formulation $\mathcal{L}({t}^{*})\mathcal{V}({t}^{*})=\mathcal{I}({t}^{*})$, where $\mathcal{V}$ and $\mathcal{I}$ are the voltage and current vectors, respectively, where elements represent the voltages and currents at the graph nodes. If all nodes of the graph are accessible and held at well-defined potentials ($n=N$), $\mathcal{L}(t)$ coincides with the memristive matrix $\mathcal{M}$ described in Eq. (5) (*i.e.*, when all nodes have well-defined potentials, $\mathcal{L}(t)$ cannot be further reduced). Conversely, the memristive matrix $\mathcal{M}$ can be interpreted as the Laplacian matrix $\mathcal{L}$ after application of the Schur complement (also known as Kron reduction). Indeed, the Kron reduction allows the Laplacian matrix to be reduced to the subset of $n$ accessible nodes with well-defined (non-floating) potentials^45^.

The *memristive (or memductive) distance* between nodes $i$ and *j* can be used to describe the evolution of the effective resistance between these nodes over time when the system is externally stimulated, provided that all other terminals are left floating. This effective resistance corresponds to the resistance distance in graph theory and to the Thevenin resistance in circuit theory. Since the concept of resistance distance^46^ parallels that of information distance^47^, the memristive distance can be interpreted as the time evolution of the information distance between the two nodes. The memristive distance between two nodes accounts not only for the shortest path length but also for the presence of multiple memristive paths between them, effectively reducing the overall distance. Therefore, it corresponds to the effective memristance, a measurable physical quantity between the two nodes.

The *transmemristance* can be evaluated according to Eq. (6) by analysing how voltage or current evolves in selected pairs of nodes that differ from the stimulated ones. As an illustrative example, we consider a memristive graph in which the dynamics of each edge are described by a potentiation-depression rate balance equation^40^, where the evolution of the internal state of an edge ($$i,j)$$ is given by:

7 $$\frac{d{x}_{ij}}{dt}={\kappa }_{P}\left(1-{x}_{ij}\right)-{\kappa }_{D}{x}_{ij}$$

where ${x}_{ij}$ is the memory state of the edge $(i,j)$, while ${\kappa }_{P}$ and ${\kappa }_{D}$ are potentiation and depression rate coefficients that exhibit an exponential dependence on the voltage difference between nodes $(i,j)$ through the physics-based relationships:

8 $$\kappa_{P} \left( {V_{ij} } \right) = \kappa_{P0} exp\left( { + \eta_{P} \left| {V_{ij} } \right|} \right),\,\,\kappa_{D} \left( {V_{ij} } \right) = \kappa_{D0} exp\left( { - \eta_{D} \left| {V_{ij} } \right|} \right)$$

where ${\kappa }_{P0},{\kappa }_{D0}>0$ and ${\eta }_{P,}{\eta }_{D}>0$ are constants. The connection between the current flowing through each edge and its corresponding voltage drop is described through the linear expression:

9 $$\left[{G}_{min}\left(1-{x}_{ij}\right)+{G}_{max}{x}_{ij}\right] {V}_{ij}={I}_{ij}$$

where ${G}_{min}$ and ${G}_{max}$ are minimum and maximum edge conductance values. Equation (9) naturally provides the definition of ${x}_{ij}$ as the normalized conductance of the edge $(i,j)$. Constants and transition rates used in this work are reported in Supplementary Table 1. In this example, it is worth noting that the nonlinear response of the internal state of the memristive edges is captured by Eqs. (7) and (8). Conversely, the linear relationship between the measurable quantities ${I}_{ij}$ and ${V}_{ij}$ (Eq. (9)) represents a first-order approximation valid in the low voltage regime, as commonly employed when modelling conventional two-terminal memristive devices^48^. This approximation is sufficient to emphasize the role of connectivity and graph-theoretical properties in the evolution of multiterminal systems, while the nonlinear dynamics remain encoded in the function $\mathcal{F}$. This approach preserves generality: it accommodates both simplified linearized models for analytical tractability and more detailed nonlinear models when the focus is on reproducing device-level behaviour. Note also that Eq. (7) describes the evolution of the internal state of a volatile type memristor. While, in principle, a memristive graph could also be composed of non-volatile memristive edges, it should be remarked that the reset process typically required for non-volatile operation can be hindered by the redistribution of the applied voltage throughout the system during switching events. As a consequence of this redistribution, and depending on the specific graph topology, achieving the electric field required for the reset of a memristive edge can become challenging, since the voltage at each node is not externally controlled (i.e., when non-accessible floating nodes are present). This limitation does not occur in ideal crossbar architectures (without the sneak-path problem), where each memristive element can be independently addressed and programmed, and no hidden nodes are present.

Figure 3c and d show the memristive and transmemristive behaviour of the graph presented in Fig. 3a, where the edge dynamics are governed by Eq. (7). When the memristive graph is externally stimulated with a voltage signal ${V}_{ij}$ applied between nodes $i$ and $j$ while all other electrodes are left floating, for example by applying a voltage pulse followed by a small read voltage (Fig. 3c, top panel), the current ${I}_{ij}$ flowing through the $i, j$ edge evolves according to the memristive behaviour of the system (Fig. 3c, middle panel). By monitoring the system through the stimulated nodes $i$ and $j$, it can be observed that the memristive distance between these nodes evolves over time, showing a potentiation process (resistance decrease) during the voltage pulse, followed by a spontaneous relaxation toward higher resistance values when the system is subsequently stimulated with a lower read voltage (Fig. 3c, bottom panel). By instead monitoring the system through nodes other than the directly stimulated ones, for instance, nodes $k$ and $l$, is it possible to observe that the memristive behaviour of the graph leads to a distinct evolution of the voltage difference ${V}_{kl}$ across these nodes (Fig. 3d, top panel). This response reflects the transmemristive effect, which couples the input node pair $i$,$j$ to the pair $k,$ $l$ (Fig. 3d, bottom panel).

**Fig. 4:**
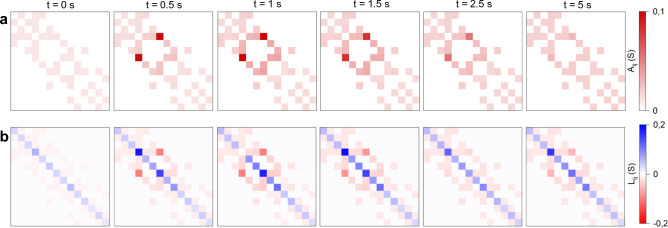
Memristive evolution of the functional connectivity of the system. Evolution over time of the (**a**). adjacency matrix and (**b**). Laplacian matrix of the memristive graph reported in Fig. 2a (according to node labelling in Supplementary Information S1).

From the above results, it becomes evident that the memristive and transmemristive behaviours of the investigated system arise directly from the evolution of the graph’s functional connectivity. This evolution can be assessed by examining the time dependence of the adjacency matrix $\mathcal{A}$, the degree matrix $\mathcal{D}$ and, consequently, the Laplacian matrix $\mathcal{L}$. Figure 4a and b shown the evolution of $\mathcal{A}$ and $\mathcal{L}$, respectively, for the graph depicted in Fig. 4a (see node labelling in Supplementary Figure S1), when the system is stimulated between nodes $i$ and $j$ using the voltage pulse shown in the top panel of Fig. 3c. Note that the degree matrix $\mathcal{D}$ is not shown, as its elements can be directly obtained from the diagonal of $\mathcal{L}$. As can be seen from the evolution of $\mathcal{A}$, the voltage stimulation between nodes $i$ and $j$ induces a modification of the system’s functional connectivity, characterized by a strengthening of specific memristive connections during the voltage pulse, followed by a progressive weakening when the smaller read voltage is applied. The strengthening of particular edges depends not only on the network topology but also on the chosen pair of stimulated nodes, which in turn influences the evolution of both $\mathcal{D}$ and $\mathcal{L}$ (Fig. 4b).

**Fig. 5:**
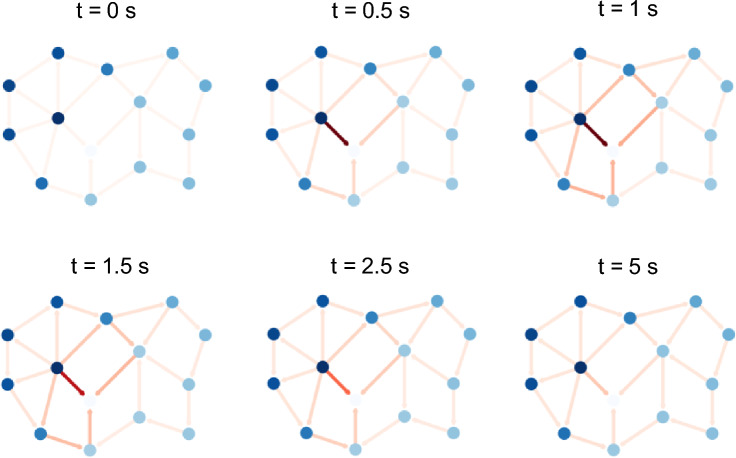
Evolution of the memristive graph. Evolution over time of the graph reported in Fig. 2a here represented as a weighted and directed graph. The red colour intensity of each edge is proportional to the edge weight (conductance) while the blue colour intensity of each node is proportional to the node voltage.

The memristive evolution of the graph can also be analysed by examining the temporal evolution of node voltages and edge currents, computed through nodal voltage analysis based on the Laplacian matrix $\mathcal{L}$. Notably, under electrical stimulation, the graph can be represented as a direct graph, where the direction of each edge indicates the corresponding current flow. Figure 5 illustrates the evolution of the graph when it is stimulated between nodes $i$ and $j$ using the voltage pulse shown in the top panel of Fig. 3c, for selected timestamps. This representation allows visualization of the evolution of the voltage distribution across all nodes, the variation of edge weights (*i.e.*, edge conductance), and the directions of edge current. In addition to the potentiation of the directly stimulated $i,j$ edge, neighbouring edges also exhibit potentiation due to the redistribution of voltage drops throughout the graph. As a result of the external stimulation, it becomes evident that specific conductive channels emerge, whose formation depend both on the choice of input nodes and on the applied voltage waveform.

### Experimental multiterminal memristive system

In practice, multiterminal memristive systems can be experimentally realized using self-organizing nanomaterial networks that exhibit memristive interactions between their nanoscale components. In this context, multiterminal systems based on nanowire networks^24,31,49–51^, nanoparticle networks^14,23,52,53^, and atomic switch networks^25,54^ have been successfully fabricated. These complex dynamical systems display emergent spatiotemporal behaviour arising from the mutual interaction among nanoscale memristive components, which can also be modeled within the framework of graph theory.^44^ In such a representation, nanoscale components correspond to network nodes, while their hysteretic interactions are represented as memristive edges. Alternatively, these systems can be viewed as coarse-grained graphs, where nodes represent entire regions and the memristive edges describe the effective interactions between these regions through a parcellation approach.^41^.

The self-organizing network can be experimentally accessed through multiple metal electrodes (accessible terminals), each typically contacting one or, more often, a cluster of nanoscale components. The nanoscale components contacted by electrodes correspond to the accessible nodes of the network, whereas components that are not directly contacted act as internal nodes of the system.

**Fig. 6:**
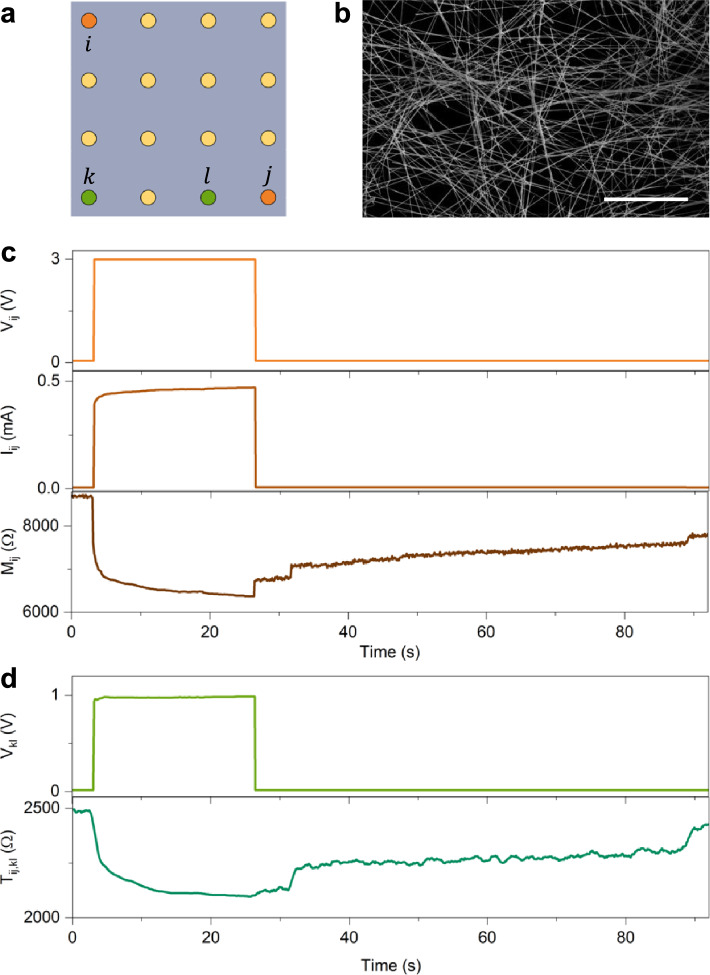
Multiterminal nanonetworks as transmemristive systems. (**a**). Example of an experimental multiterminal memristive system realized by contacting a self-organizing memristive nanowire network with metal electrodes disposed in a grid-like fashion. Here, orange and green terminals are exploited for evaluating memristive and transmemristive properties of the system. (**b**). Representative image of the self-organizing memristive nanowire network acquired through scanning electron microscopy (scale bar, 10 µm). (**c**). Input pulse voltage applied in between $i$ and $j$ terminals (top panel), corresponding evolution over time of the current ${I}_{ij}$ flowing through the memristive system (middle panel), and experimental evolution over time of the internal state of resistance (memristance). d. Experimental evolution of the voltage difference in between $k$ and $l$ terminals during stimulation between $i$ and $j$ terminals (top panel), and corresponding evolution of the transresistance over time (transmemristance) (bottom panel).

A schematic of an experimental multiterminal memristive system based on neuromorphic nanowire networks is shown in Fig. 6a, where the memristive network is contacted by metal electrodes arranged in a grid-like configuration representing the system’s accessible terminals (details of the experimental setup in Ref.^55^). A representative image of a self-organizing memristive nanowire network is shown in Fig. 6b, where the emerging volatile memristive behaviour, characterized by short-term memory, has been demonstrated to arise from resistive switching events occurring in nanowires and junctions^31,56^.

As expected, the memristive and transmemristive behaviours are a direct consequence of the system’s evolution under external stimulation. In this case, the internal (physical) state of the system, ${\boldsymbol{x}}$, can be associated with the network conductivity map (experimentally measurable through electrical resistance tomography^57,58^), which dynamically evolves depending on the spatial location and temporal sequence of input signals^32^. Fig. 6c and d show the experimental memristive and transmemristive behaviours of the multiterminal memristive system depicted in Fig. 6a. When the system is externally stimulated with a voltage signal ${V}_{ij}$ between terminals $i$ and $j$, for instance, a voltage pulse followed by a small read voltage (Fig. 6c, top panel), the resulting current ${I}_{ij}$ evolves due to the system’s intrinsic memristive dynamics (Fig. 6c, middle panel). In analogy with the memristive graph described in Sect. 5, the evolution of the system observed through stimulated $i$ and $j$ terminals exhibits potentiation (a decrease in resistance) during the voltage pulse, followed by spontaneous relaxation when the system is subsequently sensed with a small read voltage (Fig. 6c, bottom panel). In this case, stimulus-dependent deterministic dynamics coexists with stochastic effects (noise and jumps), as discussed in Ref.^59^.

Similarly to the memristive graph, analysing the system’s evolution through terminals other than the stimulated ones, for instance, terminals $k$ and $l$, reveals that the memristive behaviour gives rise to a specific evolution of the voltage difference ${V}_{kl}$ across these terminals (Fig. 6d, top panel) reflecting the transmemristive coupling between input pair $i,j$ and the output pair $k,l$ (Fig. 6d, bottom panel).

## Discussion

The extension of the two-terminal memristive concept to a general multiterminal memristive system can be obtained by replacing the scalar memristive function (denoted as *g* in Ref.^33^) with the memristive matrix formulation introduced in Eqs. (4) and (5). Importantly, this matrix is not a simple collection of individual memristances; rather, it encodes information about both the dynamics and the connectivity of the underlying memristive network or material. This framework is not limited to networks of discrete memristive devices but can also be applied to continuous materials — or materials that can be approximated as nearly continuous media — where the local conductivity depends on the spatiotemporal history of electrical stimulation. When the topology of the memristive network is known, one can explicitly determine how the elements of the memristive matrix depend on the behaviour of each memristive component. In this case, the system can be represented as a *memristive graph*, where the Laplacian matrix and its properties (e.g., eigenvalues) provide valuable insights into system connectivity, as shown in Sect. 5 of our work. Conversely, when the internal topology of the memristive network or material is not directly accessible, as often occurs in experimentally realized self-organizing memristive nanonetworks, where probing individual elements is practically unfeasible, the elements of the memristive matrix can only be determined empirically, as discussed in Sect. 6. In this latter situation, the memristive matrix serves as a powerful effective descriptor, capturing the global dynamical properties of the system without requiring explicit knowledge of its internal structure. In this framework, the concept of transmemristance provides direct insight into the coupling between different ports. This perspective not only facilitates the experimental characterization of complex self-organizing systems but also provides a conceptual bridge between nano/microscopic device physics and macroscopic functional behaviour, enabling applications in adaptive electronics, neuromorphic computation, and network-based information processing, as discussed in the following.

(i) The framework presented here establishes a connection between multiterminal memristive systems, complex networks, and graph theory. In this context, we show that the evolution of memristive systems that can be represented as memristive graphs depends on the evolution of the Laplacian matrix describing the system. Since the Laplacian matrix is the mathematical object that encodes many functional properties of a graph, the proposed framework can be used to further investigate the relationship between functional and structural connectivity in memristive networks with a network science approach. In particular, the Laplacian matrix represents the network kernel encoding the entire topology and connectivity as well as it is directly related to the modes of information propagation through the network (*i.e.*, each eigenvector corresponds to a specific transmission mode).
(ii) Analyzing the system’s evolution through multiple terminals spatially distributed across the memristive material or network provides information about the local dynamics of multiterminal memristive systems. For example, adopting a multiterminal approach, where the system is simultaneously observed from different perspectives, enables experimental investigation of the network’s local activity and its influence on the system’s computing capabilities. This approach has revealed that interactions among different regions of the network exhibit specific activation properties, as discussed in Ref.^51^.
(iii) The introduction of the concept of transmemristance enables new characterization techniques for multiterminal memristive systems. Observing the system’s evolution from multiple perspectives provides valuable insights into its internal state and underlying physicochemical mechanisms. For instance, measuring the time evolution of the transresistance of a multiterminal system under external stimulation (i.e., the transmemristance) allows one to quantitatively reconstruct the evolution of the conductivity map of self-organizing memristive systems, as demonstrated in Refs.^32^ and^58^. Notably, the transconductance represents a projection of the network kernel (Laplacian matrix) that captures how inputs drive outputs once certain terminals are fixed.
(iv) Memristance and transmemristance in multiterminal memristive systems represent physical observables correlated with the system’s dynamical internal state. These observables can be exploited for the implementation of unconventional computing paradigms, such as reservoir computing. Within unconventional computing frameworks, memristance (or memductance) and transmemristance (or transmemductance) can be employed as measurable quantities directly linked to the internal state of the physical reservoir system for the realization of novel memristive computing architectures beyond crossbar arrays.

## Conclusions

In summary, we have shown how the concept of two-terminal memristive devices can be extended to multiterminal memristive systems, in which the evolution of internal resistance states depends on the history of spatiotemporal stimulation. While the notion of memristance can be defined between any pair of terminals in such systems, a multiterminal configuration enables the tracking of internal state evolution from multiple perspectives — that is, by monitoring the system through terminals not directly involved in the stimulation. This leads to the definition of transmemristance (and transmemductance), which, as demonstrated through both a theoretical memristive graph and an experimental multiterminal memristive system, captures the relationship between the response observed at non-stimulated terminals and the input applied at the stimulated ones. The theoretical framework presented here allows different classes of multiterminal memristive systems and architectures, including crossbar arrays and self-organizing memristive networks, to be described within a unified mathematical formalism. This unified approach facilitates comparisons, generalizations, and the extension of concepts originally developed for two-terminal devices to more complex multiterminal architectures.

## Supplementary Information

Supplementary Information.

## Data Availability

All data generated or analysed during this study are included in this published article (and its Supplementary Information files).
