# Supplementary material for: Memristance and transmemristance in multiterminal memristive systems
Source: Sci Rep. 2026 Jan 14;16:5271. doi: 10.1038/s41598-026-35671-7 (PMC12881554; doi:10.1038/s41598-026-35671-7)
Supplement: Supplementary file 1 — Supplementary Information. [file 41598_2026_35671_MOESM1_ESM.docx]

**Memristance and transmemristance in multiterminal memristive systems – Supplementary Information**

*Gianluca Milano**^1*^, Davide Pilati^1,2^, Fabio Michieletti^1,2^, Alessandro Cultrera^3^, Carlo Ricciardi^2^, Enrique Miranda^4^*

^1^Advanced Materials Metrology and Life Sciences Division, INRiM (Istituto Nazionale di Ricerca Metrologica), Strada delle Cacce 91, 10135 Torino, Italy.

^2^Department of Applied Science and Technology, Politecnico di Torino, C.so Duca degli Abruzzi 24, 10129 Torino, Italy.

^3^Quantum Metrology and Nanotechnologies Division, INRiM (Istituto Nazionale di Ricerca Metrologica), Strada delle Cacce 91, 10135 Torino, Italy.

^4^Departament d’Enginyeria Electrònica, Universitat Autònoma de Barcelona (UAB), 08193 Cerdanyola del Vallès, Spain

*Corresponding author: Gianluca Milano

Email: [g.milano@inrim.it](mailto:g.milano@inrim.it)

**
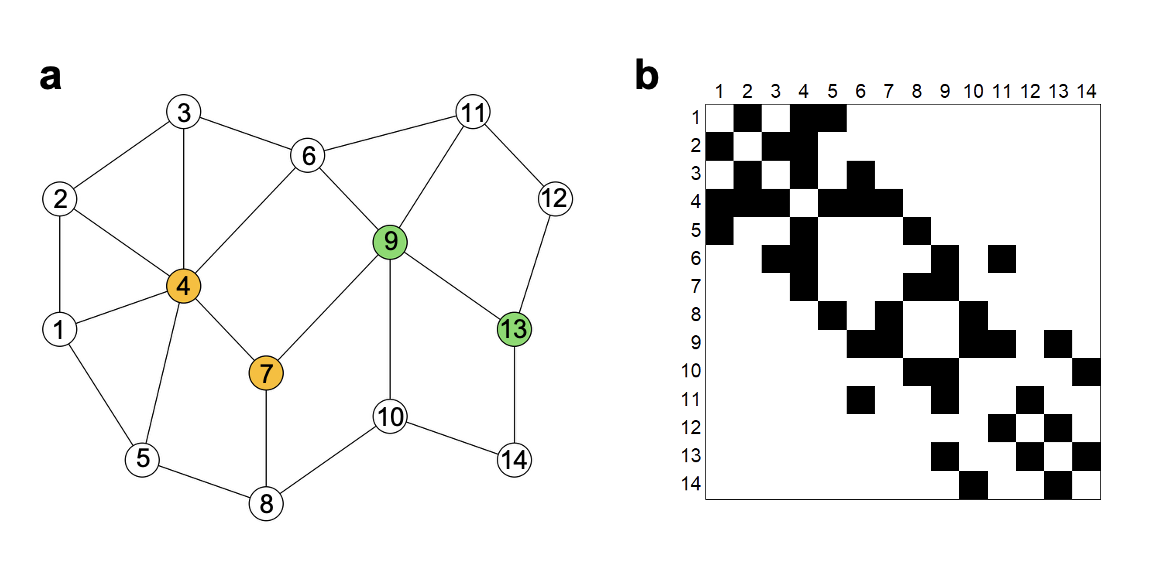
**

**Supplementary Figure S1. Node labelling. a.** Graph reported in Figure 2a with labelled nodes and **b.** corresponding adjacency matrix (unweighted) of the graph according to node labelling. The here reported the node labelling scheme that has been exploited in the adjacency matrices and Laplacian matrices reported in Figure 3.

**Supplementary Table 1.** Parameters that regulate the evolution of the conductivity of each edge of the grid graph model.

| **Parameters** | **Value** |
| --- | --- |
| $G_{\min}$ | 0.01 S |
| $G_{\max}$ | 0.1 S |
| $\eta_{P}$ | 1.8 V^-1^ |
| $\eta_{D}$ | 3 V^-1^ |
| $\kappa_{P0}$ | 0.05 s^-1^ |
| $\kappa_{D0}$ | 0.5 s^-1^ |
